# Isolation of Novel *Afipia septicemium* and Identification of Previously Unknown Bacteria *Bradyrhizobium* sp. OHSU_III from Blood of Patients with Poorly Defined Illnesses

**DOI:** 10.1371/journal.pone.0076142

**Published:** 2013-10-14

**Authors:** Shyh-Ching Lo, Guo-Chiuan Hung, Bingjie Li, Haiyan Lei, Tianwei Li, Kenjiro Nagamine, Jing Zhang, Shien Tsai, Richard Bryant

**Affiliations:** 1 Tissue Microbiology Laboratory, Division of Cellular and Gene Therapies, Office of Cellular, Tissue and Gene Therapies, Center for Biologics Evaluation and Research, Food and Drug Administration, Bethesda, Maryland, United States of America; 2 Department of Infectious Diseases, Oregon Health and Science University, Portland, Oregon, United States of America; Beijing Institute of Microbiology and Epidemiology, China

## Abstract

Cultures previously set up for isolation of mycoplasmal agents from blood of patients with poorly-defined illnesses, although not yielding positive results, were cryopreserved because of suspicion of having low numbers of unknown microbes living in an inactive state in the broth. We re-initiated a set of 3 cultures for analysis of the "uncultivable" or poorly-grown microbes using NGS technology. Broth of cultures from 3 blood samples, submitted from OHSU between 2000 and 2004, were inoculated into culture flasks containing fresh modified SP4 medium and kept at room temperature (RT), 30°C and 35°C. The cultures showing evidence of microbial growth were expanded and subjected to DNA analysis by genomic sequencing using Illumina MiSeq. Two of the 3 re-initiated blood cultures kept at RT after 7–8 weeks showed evidence of microbial growth that gradually reached into a cell density with detectable turbidity. The microbes in the broth when streaked on SP4 agar plates produced microscopic colonies in ∼ 2 weeks. Genomic studies revealed that the microbes isolated from the 2 blood cultures were a novel *Afipia* species, tentatively named *Afipia septicemium*. Microbes in the 3^rd^ culture (OHSU_III) kept at RT had a limited level of growth and could not reach a plateau with high cell density. Genomic sequencing identified the microbe in the culture as a previously unknown species of *Bradyrhizobium* bacteria. This study reports on the isolation of novel *Afipia* and *Bradyrhizobium* species. Isolation of *Bradyrhizobium* species bacteria has never been reported in humans. The study also reveals a previously unrecognized nature of hematogenous infections by the 2 unique groups of *Bradyrhizobiaceae*. Our studies show that improvement of culture system plus effective use of NGS technology can facilitate findings of infections by unusual microbes in patients having poorly-defined, sometimes mysterious illnesses.

## Introduction

Before disestablishment of the Armed Forces Institute of Pathology (AFIP), patient samples from nationwide sources were submitted to the Institute for diagnostic consultations [1]. Blood samples from patients suspected of having an infection despite of negative clinical work-ups were often sent to the Laboratory of Infectious Diseases Pathology at AFIP to rule out possible mycoplasmal infections [2]–[4]. Because mycoplasma(s) usually grow slowly in culture when first isolated from clinical specimens [5], [6], SP4 broth cultures set up for mycoplasmal isolation are often followed closely for 2–3 months. During these follow-up over months, although no microbial agent could be isolated or detected using specific PCR assays, low numbers of unknown microbes living in an inactive state in the culture broths were sometimes suspected during weekly microscopic examination. Since the microbes in question could not be effectively grown at concentrations high enough for a confirmatory study, we cryopreserved aliquots of broths from these cultures at the end of the diagnostic study for future analysis.

For a significant percentage of patients with encephalitis, pneumonitis, enterocolitis or recurrent flu-like illnesses an etiological agent has never been identified. Also, if conventional infectious disease work-ups using microbial culture systems fail to yield any positive results, the cause of the disease remains unknown and thus it is often considered to be of “viral” origin. However, the standard culture systems used might not be optimal for isolation or detection of certain microbial pathogens that live in an inactive state and are refractory to standard laboratory culture conditions. The newly developed next generation sequencing (NGS) technology offers a potential approach for analyzing every DNA molecule in an examined sample [7], [8]. This allows the analysis of nucleic acid sequences at the genome level for microbes that cannot actively multiply or grow in culture. This powerful new sequencing technology has prompted us to examine some of the previously “unresolved” microbial cultures.

This report describes the study using NGS of re-initiated cultures derived from cryopreserved culture broths of 3 blood samples from patients with various poorly defined illnesses, submitted separately between 2000 and 2004 from the Oregon Health and Science University (OHSU).

## Materials and Methods

The study conducted at FDA using previously frozen blood samples was reviewed by Department of Health and Human Services, Food and Drug Administration, Research Involving Human Subjects Committee (RISHC Protocol # 10-008B entitled "Detection of Infectious Agents in Previously Frozen Blood Samples from Patients with Various Illnesses and Healthy Blood Donors"). The original clinical presentations of patients were provided by Dr. Bryant in this study after the IRB of OHSU reviewed and determined that the activity did not meet the definition of human subject research per 45 CFR 46.102(d), and therefore written informed consent from the participants were waived. The clinical illnesses were described in the study without revealing patients' identities. The patients were lost to follow up.

### Samples, patients and clinical presentations

Dr. Richard Bryant of OHSU submitted several blood samples to AFIP for diagnostic consultations from 1999 to 2004. The samples were from patients with poorly defined illnesses who were suspected to have an infectious process despite negative infectious disease work-ups at other sites. The three blood samples analyzed in this study were received between 2000 and 2004, originally set up for isolation of mycoplasmal agents using SP4 broth medium [3], [5].

The first sample (OHSU_I) was derived from a 65 year-old Caucasian male whose chief complains were fatigue, memory loss and rapid heart rate. He had a history of rheumatoid arthritis that was treated with methotrexate and gold therapy. Serological studies for Lyme disease, Babesiosis, human granulocytic Ehrlichiosis and *M. fermentans* were all negative. The patient received combined therapy with minocycline and azithromycin which he considered helpful. The patient was referred to rheumatology but lost to follow up.

The second sample (OHSU_II) was from a 43 year-old Caucasian female with an 8-year history of post-exertion fatigue, weakness, myalgia, arthralgia, recurrent sore throat and memory impairment. Her most prominent symptoms were sore throat and drenching night sweats. She underwent a tonsillectomy seven years previously with a modest improvement in her pharyngeal symptoms and frequency of night sweats. She described non-restorative sleep but had not been observed to have sleep apnea. Serology studies against infectious agents including HIV were negative. Tender point examination substantiated diagnosis of fibromyalgia. The patient did not return to clinic and was lost to follow up.

The third sample (OHSU_III) was from a 45 year-old Caucasian male who had been a vigorously active athlete until the acute onset of malaise, muscle fasciculation and fatigue one year previously. He described recurrent myalgia, hoarseness, sore throat and recurrent flu-like symptoms that caused him give up his exercises. He also described sensations of clumsiness, blurred vision, the need to concentrate so as to speak or write coherently and symptoms of mild dysphagia. The family had a number of pets including turtles, a tortoise and a large Australian lizard. He had normal CD4 and CD8 T cell counts. Serology for Lyme disease, *Coxiella burnetti*, *Toxoplasma gondii* and *Chlamydia pneumoniae* were negative. An empirical course of minocycline had previously been unsuccessful and complicated by marked eosinophilia and symptoms of drug intolerance. The patient was an astute observer of factors affecting his health and felt that passage of time and dietary modification might have played an important part in eventually restoring his health.

### Broth Medium and agar plates

Preparation of SP4 medium (without adding antibiotics) was described previously in detail [5]. The modified SP4 medium used in this study containing 10% irradiated fetal bovine serum (FBS) and was supplemented with B12 (20 µg/L), CKM (CaCl_2_ 25 mg/L, KNO_3_ 25 mg/L, MgSO_4_ 75 mg/L) and NAD/NADP (10 mg/L). BHI and YM broths (BD, Diagnostics, Circle Sparks, MD) were prepared according to the instructions provided by the manufacturers. SP4, BHI and YM agar plates were prepared with in 1.5% Noble agar (Gibco, Grand Island, NY).

### Microbial cultures

Cryopreserved SP4 broth (100 to 200 µL) containing cultures established from blood samples were inoculated into 25 cm^2^ tissue culture flasks containing ∼ 7 mL of modified SP4 medium supplemented with 10% irradiated fetal bovine serum (FBS). Three sets of cultures were kept separately at RT, 30°C and 35°C. For each set of cultures, at least 2 control cultures of previously frozen plasma of healthy blood donors containing the same broth medium and FBS without inoculation of the cryopreserved broth were included and studied in parallel. The broths in culture flasks were checked for color changes and examined for evidence of microbial growth by inverted phase microscopy 2 to 3 times a week. The cultures showing evidence of microbial growth were diluted 1∶2 or 1∶3 with fresh modified SP4 medium and expanded into new culture flasks. Aliquots of broth (∼ 100 µL) from cultures with possible microbial growth were also streaked out on 1% agar plates using SP4, BHI or YM broth as well as TSA- 5% sheep blood agar plates (Remel Inc., Lenexa, KS). Several sets for each agar plate were kept at 3 different temperatures and examined weekly for evidence of microbial colony formation.

### Biochemical and metabolic studies using Bio-Log ID system

Biochemical reactions were performed by first growing the testing bacteria on YM agar or BCYE agar (Remel Inc., Lenexa, KS) at 30°C for a week. The test bacteria, after inoculation into GENIII testing MicroPlate (Biolog Inc, Hayward, CA), were grown at 30°C and followed for 5 to 10 days. Reading of the reaction results was conducted according to the manufacturer’s instructions using the Biology’s Microbial Identification Systems. Nitrate reductase and oxidase activities tests as well as the catalase tests using 3% hydrogen peroxide were conducted as previously described [9].

### Whole-genome/unbiased sequencing

Genomic DNA was isolated from bacteria concentrated from culture broths by super-speed centrifugation at 17K rpm for 90 min. Genomic sequencing was conducted using the MiSeq platform (Illumina, San Diego, CA). For each culture studied, 50 ng of purified DNA was subjected to DNA library construction using the Nextera DNA Sample Prep Kit (Illumina) with multiplex indexing according to the manufacturer’s protocol. A mixture of 4 to 6 separate DNA libraries was usually analyzed for each run of 2×250 bp pair-end sequencing. The sorted sequencing reads according to the unique index of each sample were assembled into contigs using CLC bio Genomics Workbench version 6.0 (Aarhus, Denmark) using the *De Novo* assembly method, after the low-quality reads were filtered out and trimmed. The GenBank Accession Numbers for OHSU_I uncloned, OHSU_I C4, OHSU_I C6, OHSU_II uncloned, OHSU_II C1, OHSU_II C2 and OHSU_III are APJG00000000, APJF00000000, APJE00000000, APHQ00000000, APJI00000000, APJH00000000 and APJD00000000, respectively.

### 16S and rRNA operon comparison and phylogenetic analysis

The complete rRNA operon (5,378 bp) of *Bradyrhizobiaceae bacterium* SG-6C (GenBank accession number GU324241) was used as query to determine the boundaries of the 16S sequences and rRNA operons for *Afipia septicemium* OHSU_II, *Afipia septicemium* OHSU_I, *Bradyrhizobium* OHSU_III, other established *Afipia* species, and some closely-related *Bradyrhizobiaceae* species. Highly similar 16S rRNA sequences from uncultured *Afipia* species were downloaded from the non-redundant database of NCBI for comparison. An overall phylogenetic relatedness among species was inferred using a Neighbor-Joining algorithm with a bootstrap analysis of 500 replicates based on the alignments of 16S rRNA and rRNA operon sequences, respectively using MEGA4 [10].

### Construction of draft genomes and genome content comparison

Alignment of the formed contigs synthesized from genomic sequencing into draft genomes using a complete bacterial genome as the reference was conducted using the CONTIGuator program [11] with the default parameters and the Blast e-value set at 1e^−5^. The complete genome of *Bradyrhizobiaceae bacterium* strain SG-6C (GenBank accession number CM001195) and the 3 contigs of *A. broomeae* (GenBank accession numbers NZ_KB375282-4) were used as reference genomes in draft genome construction for isolates of OHSU_II and OHSU_I cultures. The complete genome of *B. japonicum* USDA 110 (GenBank accession number NC_004463.1) was used as a reference genome for strain OHSU_III draft genome construction. Schematic sequence alignment for all input draft genome sequences and comparison of dissimilarity for genome contents among the related microbes were performed using progressiveMauve [12].

## Results

### Growth and isolation of microbes from cryopreserved cultures derived from 3 blood samples

The re-initiated blood cultures using modified SP4 broth kept separately at room temperature (RT, ∼ 25°C), 30°C and 35°C initially showed no sign of microbial growth. The broth streaked on SP4-agar and BHI-agar plates also produced no colonies. However, after 7 to 8 weeks, cultures from 2 patients (OHSU_I and OHSU_II) kept at RT appeared to have an increase in the number of unknown microbes during weekly microscopic examination. After a 1:3 dilutions with fresh modified SP4 medium, the microbes grew gradually reaching a plateau of higher cell density with low, but detectable turbidity after 3 to 4 weeks. None of the control cultures and the cultures kept at the higher temperatures showed similar signs of microbial growth. Gram staining revealed that the microbes in both cultures kept at RT were Gram-negative rods. Also, at least some of the microbes were evidently motile.

**Figure 1:**
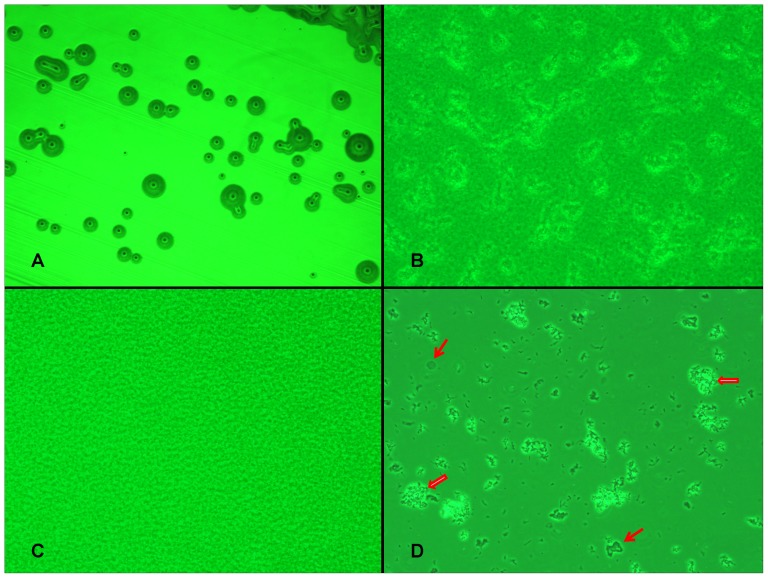
Photomicrographs of OHSU_I, OHSU_II and OHSU_III microbes. **A:** Photomicrograph of OHSU_II C1 forming microscopic colonies on the surface of a YM agar plate after 2 weeks of incubation at 30°C. The streaked lines on the agar plate could be clearly seen. 40X magnification. **B and C:** Photomicrographs of OHSU_I C6 and OHSU_II C1 microbes growing at high cell density in the culture broths kept at RT after more than 10 days. The microbes stayed at the bottom of undisturbed culture flasks. Many of the OHSU_I C6 microbes aggregated into clumps and adhered on the plastic flask surface (B). OHSU_II C1 did not aggregate into clumps or adhere on the plastic surface (C). Phase contrast with green filter 400X. **D:** Photomicrograph of OHSU_III culture. The microbes in the broth appeared to increase in numbers and form loose aggregations (empty arrows) seen at the bottom of the culture flask. However, the microbes could not grow and reach into a high cell density after 3 months of incubation at RT. Fragments of potential blood cells debris were also seen (arrows). Phase contrast 400X

The broths of both OHSU_I and OHSU_II cultures were then streaked on 1.5% Noble agar plates prepared using modified SP4 medium. Colonies were detected microscopically after 1–2 weeks on the SP4-agar plates kept at RT. The colonies remained microscopic in size after more than 3 weeks of incubation at RT. Single colonies were picked from the agar plates and grown individually in fresh modified SP4 broth medium for further studies. The single-colony derived microbes could also grow in YM broth and BHI broth without serum supplement. They formed microscopic colonies on YM agar (Figure 1A), BCYE agar and TSA-sheep blood agar kept at 30°C and 35°C (Table 1). Interestingly, most of the microbes could grow at 37°C in a CO_2_ incubator on agar plates made using tissue culture medium RPMI-1640 with 5% fetal bovine serum. But, they did not grow on MacConkey agar kept at any temperature. Compared to the microbes from OHSU_II cultures, the microbes from OHSU_I cultures showed a higher tendency to adhere onto plastic surfaces and form clumps of consisting of bacterial aggregations (Figure 1, B and C).

**Table 1:** Comparison of growth characteristics, biochemical reactions and G/C contents of *Afipia* species.

Growth Characteristics†	1	2	3	4	5	6	7	8	9	10	11	12	13	14	15	16
**Motility:**	p	N	N	p	p	p	P	P	P	P	P	P	P	P	N	N
**Growth on:**																
** BCYE agar (RT)**	P	P	P	P	P	P										
** 30°C**	P	P	P	P	P	P										
** 35°C**	P	P	P	p	p	p	P	P	p	P	P	P	N	P	N	N
** 37°C**	p	P	p	p	p	p	P	P	N	P	P	P	N	P	N	N
** YM agar (RT)**	P	P	P	P	P	P										
** 30°C**	P	P	P	P	P	P										
** 35°C**	p	P	p	p	p	p										
** 37°C**	p	p	p	p	p	p										
** BHI agar (RT)**	p	p	p	p	p	p										
** 30°C**	p	p	p	p	p	p										
** 35°C**	N	N	N	N	N	N										
** Columbia agar_5% sheep blood (30°C)**	p	p	p	p	p	p	p	p	P	N	N	p	p	N	N	N
** TSA_5% sheep blood agar (RT)**	p	p	p	p	p	p										
** 30°C**	p	p	p	p	p	p										
** 35°C**	p	p	p	p	p	p										
** 37°C**	p	p	p	p	p	p										
** RPMI-5% FBS 35°C/CO2 incubator**	p	P	p	p	P	P										
** 37°C/CO2 incubator**	p	p	p	P	P	P										
** MacConkey agar (RT)**	N	N	N	N	N	N										
** 30°C**	N	N	N	N	N	N	N	N	p	N	N	N	N	N	N	N
** 35°C**	N	N	N	N	N	N										
**Catalase**	p	p	p	p	p	p										
**Oxidase**	P	P	P	P	P	P										
**Nitrate reduction**	p	p	p	P	P	p	P	P	N	N	N	N	P	N	P	P
**G+C content (mol%)**	61.1	61.1	61.1	61.1	61.1	61.1	62.5	63.1	64	69	67	65.5	61.9	61.5	59.3	60.2

Biochemical Reactions‡	1	1a*	2	2a*	3	3a*	4	4a*	5	5a*	6	6a*	7	8	9	10	11	12	13	14	15	16
**Assimilation of:**																						
** Glucose**	N	N	N	N	N	N	P	N	N	N	p	N	N	N	N	P	P	N	N	N	N	N
** Arabinose**	N	N	N	N	N	N	N	N	N	N	N	N	N	N	N	P	P	N	N	N	N	N
** Mannose**	N	N	N	N	N	N	P	N	N	N	N	N	N	N	N	P	P	N	N	N	N	N
** Mannitol**	N	N	N	N	N	N	P	N	N	N	N	N	N	N	N	P	P	N	N	N	N	N
** N-Acetylglucosamine**	N	N	N	N	N	N	P	N	N	N	N	N	N	N	N	P	P	N	N	N	N	N
** Gluconate**	N	P	N	N	P	p	P	P	P	P	P	P	N	N	P	P	P	P	N	P	N	N
** Adipate (acidity regulator)**													N	N	P	P	P	P	P	P	P	P
** Malate**	N	p	p	N	N	N	P	P	p	P	P	P	P	P	P	P	N	P	P	P	N	N
** D-Malic acid**	N	p	N	N	N	N	p	p	P	P	P	p										
** L-Malic acid**	N	p	P	N	N	N	p	p	N	p	P	p										
** Citrate**	N	N	N	N	N	N	N	N	N	N	N	N	N	N	N	P	N	N	N	N	N	N
** Phenylacetate**	P	P	P	P	P	P	N	P	P	P	P	P	N	N	N	P	N	P	N	P	N	N

† *Afipia* species and related strains or isolates: 1, OHSU_I uncloned; 2, OHSU_I-C4; 3, OHSU_I-C6; 4, OHSU_II uncloned; 5, OHSU_II-C1; 6, OHSU_II C2; 7, A. felis; 8, *A. felis* genospecies; 9-12, *Afipia* genospecies (1 to 4); 13, *A. clevelandensis*; 14, *A. broomeae* (3 strains); 15, *A. birgiae*; 16, *A. massiliensis*. Data for *A. broomeae*, *A. clevelandensis*, *A. felis*, *A. felis* genospecies, 4 *Afpia* genospecies, *A. birgiae* and *A. massiliensis* are from Brenner et al. (15) and La Scola et al. (9).

‡ Bacteria were grown on YM agar before use of biochemical studies, except noted by a* for grown on BCYE agar.

P: positive, p: weak positive, N: negative.

The re-initiated culture from a 3^rd^ patient (OHSU_III) studied in parallel also appeared to have an increase of microbial growth in flasks that were kept at RT for 3 months. Some of the “microbes” loosely aggregated without clearly adhering onto the surface of flask (Figure 1 D). However, the microbes in culture derived from the OHSU_III sample differed from those from the OHSU_II and OHSU_I samples in that they failed to continue to proliferate reaching a plateau of high cell density and no turbidity could be detected in the broth. Also, the broth streaked on SP4, YM, TSA-sheep blood, chocolate and BCYE agar plates produced no colonies.

### Biochemical characterization of blood-derived microbes using the Biolog identification system

Biochemical properties and metabolic characterization of representative single-colony cloned OHSU_II (C1 and C2) and OHSU_I (C4 and C6) bacteria were studied using Biolog bacterial identification kits. The system offers phenotypic microarrays enabling microbes to be evaluated for a host of phenotypes under many culture conditions in a rapid and cost-effective manner. Since OHSU_II and OHSU_I bacteria failed to grow on Biolog BUY and BUG agar plates, they were first grown on YM or BCYE agar plates for 7 days and then transferred to the Biolog micro-plates. The test results obtained displaying important sugar assimilations and biochemical reactions are listed in Table 1. Since the genomic studies revealed that OHSU_II and OHSU_I bacteria were most closely related to *Afipia* bacteria (see below), properties of established *Afipia* species reported in previous studies [9], [13] are also listed in Table 1 for comparison. It was noted that the un-cloned bacteria pre-grown on different kinds of agar plates produced different biochemical reaction results in some tests. The metabolic results obtained from OHSU_II and OHSU_I bacteria did not match any specific bacterium in the Biolog identification database.

### Ultrastructure study of OHSU_I and OHSU_II bacteria

**Figure 2:**
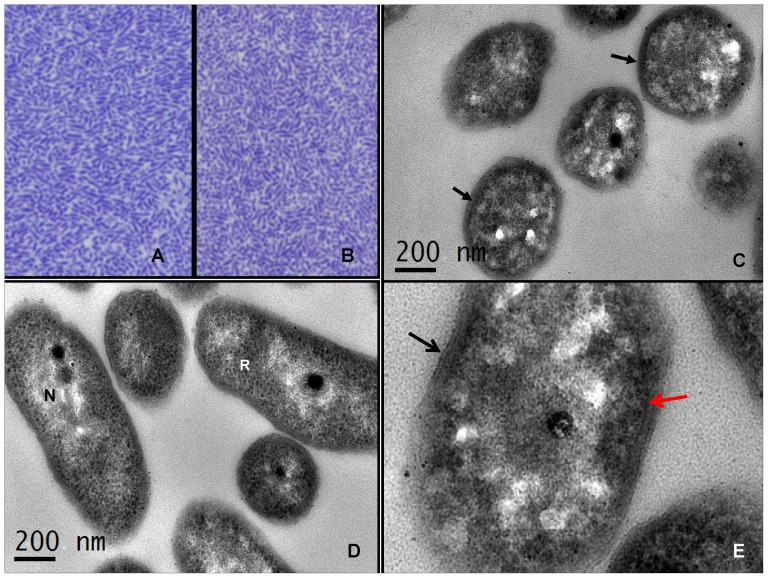
Thick section photomicrographs and ultrathin electron micrographs of OHSU_I and OHSU_II microbes. **A and B:** Thick section photomicrographs of OHSU_I C6 (A) and OHSU_II C1 (B) that were concentrated from cultures using modified SP4 broth. Both sections revealed morphologically similar, slender pointed microbes using longitudinal and cross sectioning. The concentrated microbes were fixed with 2.5% glutaraldehyde, post-fixed with 1% osmium tetroxide and embedded in epoxy resin. The thick sections were stained using 1% toluene blue. 1000X. **C and D:** Electron photomicrographs of OHSU_I C6 (C) and OHSU_II C1 (D) captured in ultrathin sections. Typical Gram-negative bacteria wall structure (arrows), intracellular ribosomal structures (R), electron-dense bodies and nucleic acid (N) as well as scale bars are indicated for both sections. The ultrathin sections were stained with uranyl acetate and lead citrate. **E:** Electron micrograph of OHSU_I C6 captured at higher magnification (200,000X). External membrane (black arrow) and internal membrane structures (red arrow) of the microbes are highlighted.

The single-colony cloned microbes OHSU_II C1 and OHSU_I C6 grown in modified SP4 broth were examined by electron microscopy (EM). Figure 2 shows thick section photomicrographs of the concentrated bacterial samples (OHSU_I C6 and OHSU_II C1) embedded in epoxy resin and electron photomicrographs of their ultra-thin sections. Although OHSU_II C1 and OHSU_I C6 bacteria demonstrated different phenotypic properties in the broth cultures (Figure 1B and 1C), the concentrated microbes in thick sections appeared to be morphologically very similar (Figures 2A and 2B). The ultra-thin sections revealed unique ribosomal structures, high electron density particles and as well as the fine internal granular structure of the microbes (Figures 2C and 2D). Typical Gram-negative bacterial wall structures with outer and inner membranes were identified for the microorganisms (Figure 2E).

### Whole-Genome sequencing of OHSU_I and OHSU_II bacteria

We performed whole-genome sequencing of the original OHSU_II and OHSU_I bacteria grown in SP4 broths and on subsequent cultures obtained using single-colony cloning referred to as OHSU_II C1, OHSU_II C2, OHSU_I C4 and OHSU_I C6 respectively. The raw reads generated for each sample were assembled into contigs using CLC bio Genomics Workbench. The sequencing data, sufficient to have more than one hundred of coverage of bacterial genomes of ∼ 5 million bp, were obtained by Illumina MiSeq for almost every culture samples examined (Table 2).

**Table 2:** Whole genome sequencing datasheet of new *Bradyrhizobiaceae* from 3 blood samples.

	*Afipia septicemium*			*Afipia septicemium*			*Bradyrhizobium sp.*
	OHSU_I	OHSU_II	OHSU_III
	uncloned	C4 clone	C6 clone	uncloned	C1 clone	C2 clone	uncloned
**No. of raw reads**	4,919,572	5,765,026	4,179,986	5,824,680	14,070,208	7,511,066	10,672,800
**Total reads length (bp)**	663,772,706	558,896,845	394,058,360	760,742,197	1,937,686,091	969,427,127	1,410,159,376
**No. of qualified reads**	4,873,362	5,516,748	4,046,682	5,714,890	13,933,514	7,382,722	10,585,984
**No. of reads in contigs**	4,622,060	5,346,223	3,923,957	5,572,318	13,815,908	7,283,321	10,466,061
**No. of contigs formed**	47	50	67	48	42	69	82
**Max contig length**	486,104	551,913	366,447	631,437	604,412	564,541	714,326
**N50**	256,163	283,242	163,895	244,190	274,283	187,761	329,545
**Total contig length**	5,083,702	5,082,513	5,091,428	5,081,292	5,084,693	5,082,998	7,935,543
**Estimated coverage**	124	106	75	146	378	188	175
**GC contents (%)**	61.1	61.1	61.1	61.1	61.1	61.1	64.7

**Figure 3:**
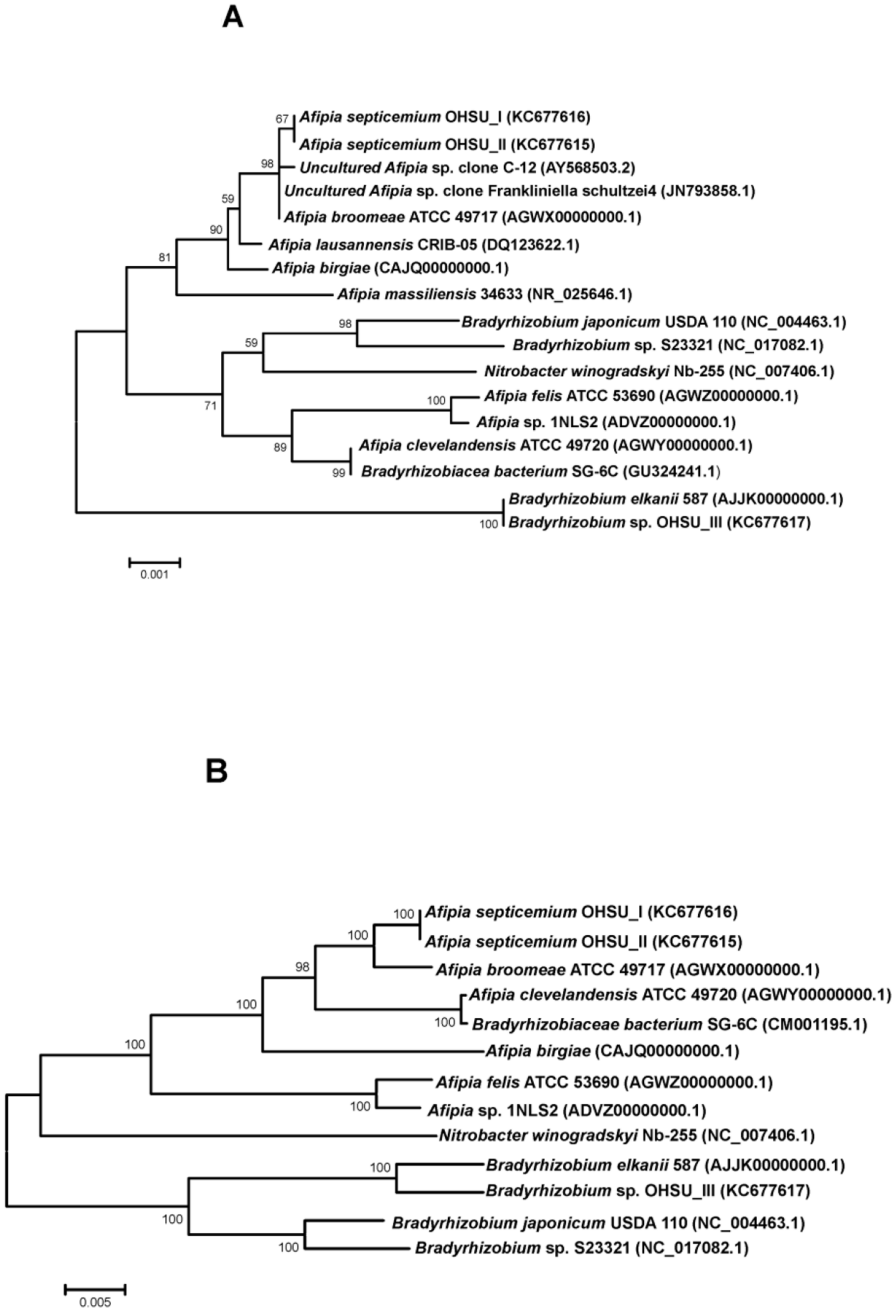
Phylogenetic relatedness of OHSU_I, OHSU_II and OHSU_III microbes among different *Bradyrhizobiaceae* species. Phylogenetic analysis based on 16S rRNA gene sequences (A) and whole rRNA operon sequences (B) using the neighbor-joining method. GenBank Accession numbers of sequences used in the analyses are shown in parentheses. Scale bar units are estimated branch lengths. Numerals indicate bootstrap percentages over 50 after 500 replications.

**Figure 4:**
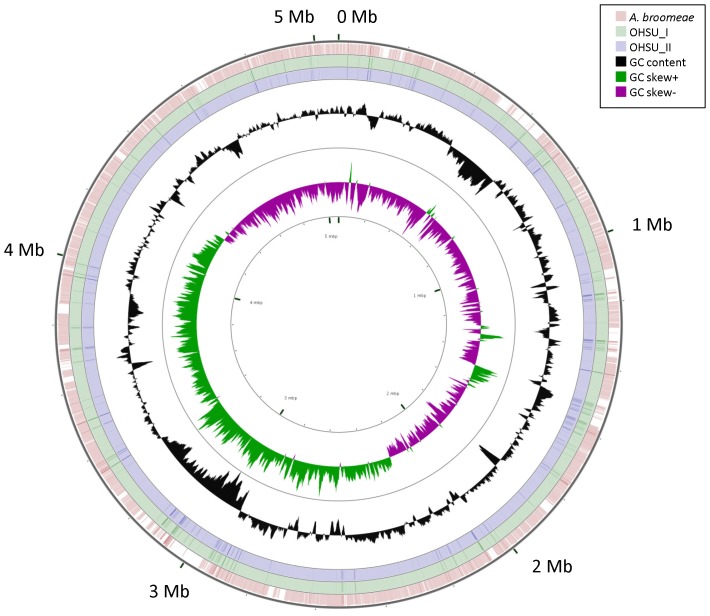
Sequence mapping for draft genomes of *A. septicemium* grown in OHSU_II and OHSU_I cultures and genome of *A. broomeae.* The complete genome of *Bradyrhizobiaceae* SG-6C was used as the reference to align genomic scaffold supercontigs 1.2, 1.3 and 1.1 of *A. broomeae* into a working complete genome that was then used as the reference genome for assembling formed contigs from genomic sequencing of the microbes grown in OHSU_I and OHSU_II cultures into draft genomes. The tracks from inside to outside represent GC skews, GC contents, draft genome of OHSU_II microbe, draft genome of OHSU_I microbe and *A. broomeae* genome. The color blank regions represent sequence differences found between the bacterial genomes. Major regions of difference are seen in the regions of 0.6 Mb and 3 Mb.

**Figure 5:**
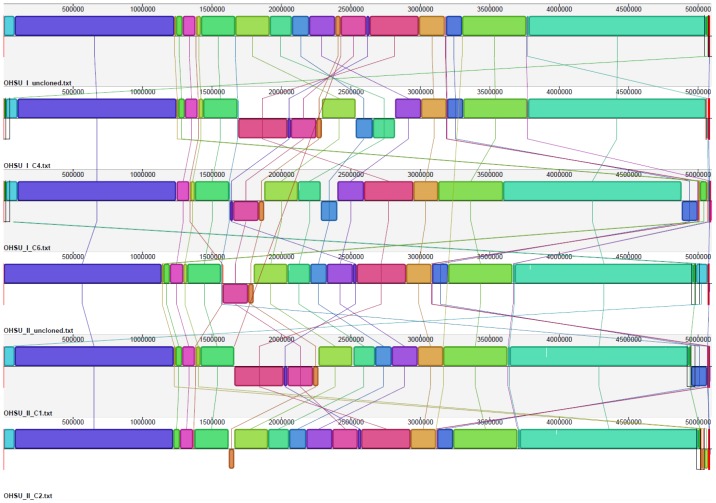
Alignment of whole-genome sequences using draft genomes of OHSU_I isolates (un-cloned, C4 and C6) and OHSU_II isolates (un-cloned, C1 and C2). Homologous locally collinear blocks among genomes are connected with a line and identified by the same color using progressiveMauve. Blocks that are inverted compared to the OHSU_I (un-cloned) genome are placed under the center line of the genome. Gene rearrangements or inversions among the genomes are indicated. The genome content dissimilarities among these isolates were calculated and are shown in Table 3.

1) **Analysis of 16S rRNA gene and rRNA operon sequences.** Comparison of sequences of 16S rRNA genes (1.48 Kb) of OHSU_I and OHSU_II with those in the NCBI database showed that the 2 isolates were a new *Bradyrhizobiaceae*, most closely related to those of *Afipia* species (Figure 3A). Since all *Bradyrhizobiaceae* have their 3 rRNA genes similarly organized and co-transcribed as an operon, analysis based on the variations in sequences of whole rRNA operon (∼ 5.4 Kb) could be more informative in the study of challenging *Bradyrhizobiaceae* taxonomy. We compared sequences of whole 16S rRNA operon for phylogenic relatedness of OHSU_I and OHSU_II isolates among the established *Afipia* sp. (Figure 3B). All isolates of OHSU_I and OHSU_II were found to have an identical ∼ 1.5 Kb rRNA gene sequence as well as an identical ∼ 5.4 Kb 16S rRNA operon sequence. Analysis of sequences of the rRNA operon revealed OHSU_I and OHSU_II microbial isolates are likely a new species of *Afipia*, tentatively named *A. septicemium* in this study. They are phylogenetically more related to *A. broomeae* than to *A. clevelandensis, A. birgiae* and *A. massiliensis.* They are most distant phylogenetically from *A. felis*.

2) **Analysis of genome sequences.** A completed genome of a newly isolated *Bradyrhizobiaceae* strain SG-6C was recently reported [14]. SG-6C was found to be closely related to *A. clevelandensis* based on both their 16S rRNA gene sequences (Figure 3) and their genome analysis (Table 3). Moreover, a draft genome of *A. broomeae* with 4 genomic scaffold supercontigs also became available [15]. We used the complete genome of SG-6C as the reference genome to construct a working complete genome of *A. broomeae* using CONTIGuator [11]. However, the complete genome of *A. broomeae* was constructed using 3 supercontigs (1.2, 1.3 and 1.1; a total of ∼ 5.1 Mb, GC content 61%) without including supercontig 1.4 (128 Kb, GC content 63%). Sequence of supercontig 1.4 could not be aligned with either SG-6C or *A. clevelandensis* genome sequences and revealed significant homology with several plasmids of alph-2 proteobacteria in Blastn search against GenBank database. The formed contigs from our genomic sequencing (Table 2) were aligned into draft genomes of the microbes grown in OHSU_II and OHSU_I cultures and the microbes of single-colony cloned from the cultures (OHSU_II C1, OHSU_II C2, OHSU_I C4 and OHSU_I C6) using the constructed genome of *A. broomeae* as reference. Figure 4 shows closely mapped *A. septicemium* and *A. broomeae* genome sequences with regions of difference identified using CGView program [16]. Contigs alignment and whole-genome sequence analysis by CONTIGuator revealed possible re-arrangements for genes ranging from ten-thousand to hundred-thousand base pairs in the microbial genomes of different colonies picked from the same culture broth (Figure 5).

**Table 3:** Genome content differences among OHSU_I, OHSU_II isolates of *A. septicemium*, established *Afipia* species and *Bradyrhizobiaceae* SG.

	1	2	3	4	5	6	7	8	9	10	11	12
**1. OHSU_I-uncloned**	-	0.06%	0.11%	0.09%	0.08%	0.06%	22.43%	30.41%	30.66%	44.91%	43.26%	30.34%
**2. OHSU_I-C4**	0.06%	-	0.10%	0.07%	0.07%	0.06%	22.39%	30.38%	30.64%	44.89%	43.24%	30.31%
**3. OHSU_I-C6**	0.11%	0.10%	-	0.12%	0.13%	0.11%	22.58%	30.52%	30.77%	45.00%	43.34%	30.45%
**4. OHSU_II-uncloned**	0.09%	0.07%	0.12%	-	0.09%	0.09%	22.42%	30.40%	30.65%	44.89%	43.25%	30.31%
**5. OHSU_II-C1**	0.08%	0.07%	0.13%	0.09%	-	0.07%	22.43%	30.41%	30.68%	44.90%	43.26%	30.34%
**6. OHSU_II-C2**	0.06%	0.06%	0.11%	0.09%	0.07%	-	22.45%	30.41%	30.64%	44.91%	43.26%	30.34%
**7. ** ***A. broomeae***	22.43%	22.39%	22.58%	22.42%	22.43%	22.45%	-	31.11%	30.67%	45.09%	44.05%	30.88%
**8. ** ***A. clevelandensis***	30.41%	30.38%	30.52%	30.40%	30.41%	30.41%	31.11%	-	32.19%	44.90%	43.87%	10.13%
**9. ** ***A. birgiae***	30.66%	30.64%	30.77%	30.65%	30.68%	30.64%	30.67%	32.19%	-	45.33%	44.49%	32.12%
**10. ** ***A. felis***	44.91%	44.89%	45.00%	44.89%	44.90%	44.91%	45.09%	44.90%	45.33%	-	27.02%	44.74%
**11. ** ***Afipia*** ** sp. 1NLS2**	43.26%	43.24%	43.34%	43.25%	43.26%	43.26%	44.05%	43.87%	44.49%	27.02%	-	43.73%
**12. ** ***Bradyrhizobiaceae*** ** SG**	30.34%	30.31%	30.45%	30.31%	30.34%	30.34%	30.88%	10.13%	32.12%	44.74%	43.73%	-

Rows 1-12 correspond to columns 1-12.

A comparison of the contents of whole genomes among OHSU_II and OHSU_I isolates as well as the established *Afipia* species for which the genome sequences are available in the NCBI database was conducted using the informatics tool progressiveMauve [12]. The comparison revealed that the 2 isolates OHSU_II and OHSU_I were indeed highly similar, with only 0.1% to 0.2% dissimilarity in content between their genomes. The microbes grown in the original un-cloned OHSU_II and OHSU_I cultures and those single-colony cloned isolates from the mother cultures all had less than 0.2% dissimilarity in content among their genomes. In comparison, genomes of OHSU_II and OHSU_I microbes had more than 22% dissimilarity in content with that of *A. broomeae* genome (Table 3) and showed even higher dissimilarity (more than 30%) with those of the *A. clevelandensis* and *A. birgiae* genomes [17]. The 2 isolates of *A. septicemium* were most different from *A. felis and A. sp* 1NLS2 with ∼ 45% dissimilarity among their genome contents.

### Whole-genome sequencing of OHSU_III culture

Unbiased deep sequencing of DNA recovered from the broth of the OHSU_III blood culture generated sequence data sufficient to have in average 175 fold coverage of a total ∼ 7.9 Mb formed contigs length (Table 2). GC content of the ∼ 7.9 Mb formed contigs was 64.7%, significantly higher than that of *A. septicemium* isolates (61.1% of ∼ 5.1 Mb formed contigs length). Only one type of 16S rRNA gene and rRNA operon were identified among contigs assembled. Analysis of 16S rRNA gene and rRNA operon sequences revealed that the microbe in the OHSU_III culture was a *Bradyrhizobium,* phylogenetically most related to *B. elkanii* (Figure 3).

**Figure 6:**
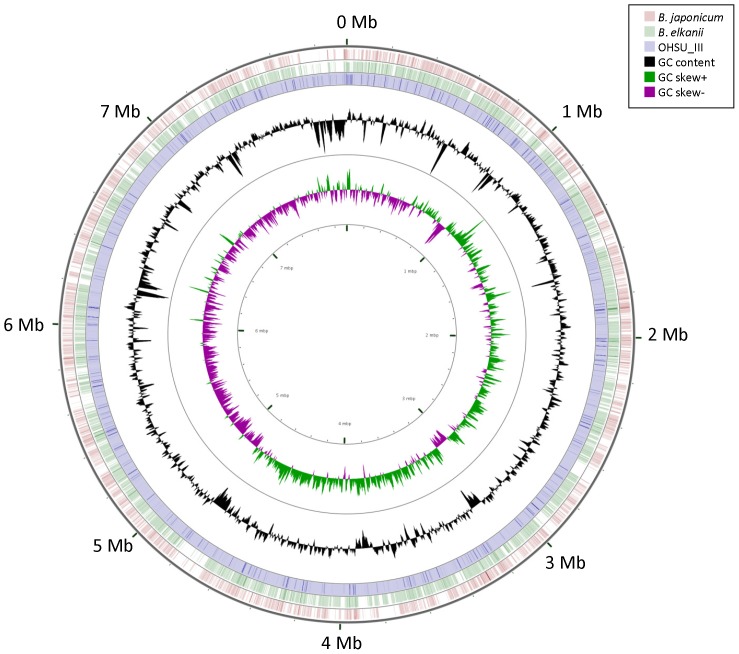
Sequence mapping for draft genomes of *Bradyrhizobium* sp. OHSU_III and the genomes of *B. elkanii* and *B. japonicum.* The tracks from inside to outside represent GC skews, GC contents, draft genome of OHSU_III microbe, draft genome *B. elkanii* and complete genome of *B. japonicum.*. The color blank regions represent areas of sequence differences found between the bacterial genomes.

Near 97.3% of total sequence length from the formed contigs of OHSU_III microbe could be aligned to genome sequences *of B. japonicum* by CONTIGuator. A draft genome of *Bradyrhizobium* strain OHSU_III was assembled by aligning the formed contigs using the complete *B. japonicum* genome of 9.2 Mb [18] as reference. Figure 6 shows sequence mapping between draft genomes of OHSU_III and genomes of *B. elkanii* or *B. japonicum* with areas of difference identified. Table 4 shows comparison of genome content for dissimilarities among genomes of *Bradyrhizobium* strain OHSU_III, *B. elkanii, B. japonicum* as well as the other established *Bradyrhizobium* with genome sequences available in the NCBI database. Strain OHSU_III appeared to be a new *Bradyrhizobium* species, clearly different from all the other established *Bradyrhizobium* species. There was nearly 40% dissimilarity in genomic content between OHSU_III and *B. elkanii* having a genome size of ∼ 8.7 Mb with 63.6% GC, despite their identical 16 S rRNA gene sequence. OHSU_III was evidently more different from *B. japonicum* with 55% dissimilarity between their genome contents (Table 4).

**Table 4:** Genome content differences among *Bradyrhizobium* sp. OHSU_III and established *Bradyrhizobium* species†.

	1	2	3	4	5	6	7
**1. B. sp. OHSU_III**	-	39.39%	55.35%	56.92%	55.96%	54.34%	57.30%
**2. B. *elkanii***	39.39%	-	54.99%	60.18%	59.06%	57.13%	59.66%
**3. B. *japonicum***	55.35%	54.99%	-	57.89%	56.45%	37.26%	40.59%
**4. B. sp. BTAi1**	56.92%	60.18%	57.89%	-	37.45%	57.01%	59.77%
**5. B. sp. ORS278**	55.96%	59.06%	56.45%	37.45%	-	55.44%	58.31%
**6. B. sp. S23321**	54.34%	57.13%	37.26%	57.01%	55.44%	-	38.41%
**7. B. sp. WSM471**	57.30%	59.66%	40.59%	59.77%	58.31%	38.41%	-

Rows 1–7 correspond to columns 1–7.

† The GenBank accession numbers for *Bradyrhizobium* species are as following: *B. elkanii* (AJJK00000000), *B. japonicum* (BA000040.2), *B*. sp. BTAi1 (CP000494.1), *B*. sp. ORS278 (CU234118.1), *B*. sp. S23321 (AP012279.1) and *B*. sp. WSM471 (CM001442.1).

## Discussion

The alpha-subdivision of the Proteobacteria is a diverse group of Gram-negative microorganisms. Some members of alphaproteobacteria such as the *Rickettsiaceae*, *Brucellaceae and Bartonellaceae* are well-known human pathogens [19], [20]. In the *Bradyrhizobiaceae*, members of *Afipia* species are also known to be pathogenic to humans [13]. *Afipia* species were found to be amoeba-resistant [21], a property allowing the microbes to survive inside macrophages. Using the amoebal co-cultivation system, La Scola et al. reported isolation of 2 species *A. birgiae* and *A. massiliensis* as well as an *A. felis* genospecies A from a hospital water supply [9]. It was hypothesized that some *Afipia* bacteria were likely causative agents of nosocomial infections. However, the apparent difficulty of isolating the microbes with traditional culture systems could render many infections un-recognized.

Because only low numbers of microbes in an inactive state were present in our earlier SP4-broth culture, we reasoned that they might require specific nutrients or suitable growth signals before they could undergo proliferation and grow to a plateau of higher cell density in culture. SP4 broth, a highly enriched medium originally developed for cultivation of spiroplasmas from infected plants or insects [22], [23], was often used in supplement with serum to facilitate isolation of fastidious human mycoplasmas [24]. The factors that could be important in helping microbes in the re-initiated blood cultures to proliferate and to reach a significantly higher cell density in the broths included adding vitamin B12, CKM and NADH/NADPH supplements in modified SP4 medium. Also, cell growth at RT was evidently crucial since the microbes in the SP4 broth cultures clearly preferred to grow at a lower temperature. All the re-initiated cultures that allowed the microbes to grow to a higher cell density were kept at RT. Some of the bacteria that did grow and reach a plateau at a higher cell density evidently gained the ability to form colonies on various agar plates kept at higher temperatures (Table 1).

Although there were only a limited number of *Afipia* isolates with complete metabolic characterization, an increasing number of 16S rRNA gene sequences for *Afipia* sp. became available in GenBank. Many of the sequences were submitted from studies involving PCR products using pan-bacteria primers targeting the 16S rRNA gene sequences. However, the microbes in this genus, or even in this family, are known to have very high levels of homology in their 16S rRNA gene sequences. They are a poor indicator to determine species or genera diversity [9], [25]. Thus, what presently classified as *Afipia* bacteria based on 16S rRNA sequences are likely to be a heterogeneous group of *Bradyrhizobiaceae*. For bacterial taxonomic analyses, 23S rRNA gene sequences, like those of 16S rRNA gene, were frequently used [26]. Since slight divergence of 16S rRNA gene sequences (∼ 1.4 Kb) could be remarkably significant in taxonomy of *Afipia* species, sequence comparison was also conducted in this study for the entire rRNA operon consisting of 16S, 5S and 23S rRNA genes (∼ 5.4 Kb) among *A. septicemium* and the established *Afipia* species. The phylogenetic analysis showed that *A. septicemium* is more closely related to *A. broomeae* than to *A. clevelandensis*, *A. birgiae* or *A. massiliensis* (Figure 3B).

The relative divergence calculated from DNA-DNA dissociation studies at different temperatures [13] or from DNA-DNA hybridizations data [9] was used previously as a powerful tool to assess the relatedness between 2 different microbes of *Afipia* or *Bradyrhizobium* species [27]. The evolving era of whole-genome sequencing has however allowed direct comparison of the content of whole-genomes to assess relatedness among a group of microbes. A comparison of the contents of whole genomes revealed that *A. broomeae, A. clevelandensis* and *A. birgiae* were about equally divergent from one another (∼ 30% dissimilarity) and more distant to *A. felis* (near 45% dissimilarity) (Table 3). Similar to the phylogenetic relatedness, *A. septicemium* was found more closely related to *A. broomeae.* However, the 2 microbes still had more than 22% of dissimilarity in genome content. The contig 4 of *A. broomeae* [15], an ∼ 128 Kb sequence, likely a plasmid, could not be found in *A. septicemium* and in any established *Afipia* species examined. Genomic and biochemical studies showed that *A. septicemium* is a novel species different from all previously known *Afipia* species. It is important to note that some *Afipia* species microorganisms were isolated previously from respiratory secretions or tissue lesions of patients [13]. However, *Afipia* bacteria have never been reported previously to have hematogenous infections in humans or isolated from blood samples of patients.

The genomic study of OHSU_III culture demonstrated that unbiased Massive Parallel Sequencing analysis of microbes that could not actively grow or multiply in culture is an effective tool. The microbe in OHSU_III culture is shown to be a previously unknown *Bradyrhizobium*. *Bradyrhizobium* are normally considered as nitrogen-fixation bacteria or bacteria having the ability of invading, surviving and symbiotically growing in the eukaryotic plant cells. To our knowledge, isolation of *Bradyrhizobium* has never been reported in humans. Certainly, no *Bradyrhizobium* has been isolated form patient blood. Our findings reveal a previously unknown host spectrum of infections by *Bradyrhizobium* bacteria. Interestingly, the sequence comparison revealed that none of the nif and nod genes or Symbiosis Islands found in genomes of *B. elkanii, B. japonicum* and other nitrogen-fixation *Bradyrhizobium* sp. isolated from soil or plants [18], [28] are present in the genome of OHSU_III. We plan to continue the cultures of the microbes that are alive but in a less-active state in OHSU_III culture. Once we are capable of more effectively growing the newly discovered *Bradyrhizobium* in the broths or clone them on agar plates, their metabolic and pathobiologic characteristics as well as drug susceptibility will be studied in detail.

Although it is still not clear if these new *Bradyrhizobiaceae* of *Rhizobiales* were the causative agents of the patients’ poorly-defined illnesses, the study has revealed a previously unrecognized nature of hematogenous infections in humans by this unique group of microbes known to be capable of intracellular growth. However, many well-known alphaproteobacteria pathogens such as *Rickettsiaceae of Rickettsiales as well as Brucellaceae and Bartonellaceae* of *Rhizobiales* are related to hematogenous infections or disseminations in humans. The poorly defined illnesses in patients having polymorphic clinical manifestations are likely to have heterogeneous causes. Our findings show some of the difficult-to-diagnose illnesses could be associated with infections of unknown *Rhizobiales*. We anticipate that improved culture systems and improved condition for isolating microbes as well as more effective use of the NGS technology will help detect these previously “uncultivated”, presumably facultative intracellular microbes in the patients. Our successful growth and isolation of these previously unknown and uncultured microbes should facilitate development of useful assays for rapid clinical diagnosis and effective analysis of pathogenicity roles these microbes may play in various human disease processes.

**Note:** While this paper is in press, we are aware that a sequence-based discovery of new *Bradyrhizobium enterica* in transplant patients with cord colitis syndrome has recently been reported [29]. The finding is important in revealing infections of the otherwise unknown and uncultured *Bradyrhizobiaceae* bacteria in patients. Analysis of 16 S rRNA gene and rRNA operon sequences shows that *Bradyrhizobium enterica* and *Bradyrhizobium* sp. OHSU-III are phylogenetically closely related. Comparison of genome sequences (NCBI accession numbers: AMFB00000000 and APJD00000000) using progressiveMauve shows the 2 new *Bradyrhizobium* microbes found infecting the human hosts are more closely related to each other than to any other established species of *Bradyrhizobium* including *B. japonicum* and *B. elkanii*. However, the 2 *Bradyrhizobium* are different and possess ∼ 30% dissimilarities between their genome contents. More novel species of *Bradyrhizobiaceae* and *Phyllobacteriaceae* microbes may likely be found in the human hosts with various clinical presentations.
